# GIS-based calculation method of surge height generated by three-dimensional landslide

**DOI:** 10.1038/s41598-023-34798-1

**Published:** 2023-05-11

**Authors:** Guo Yu, Xiaowen Zhou, Lei Bu, Chengfeng Wang, Asim Farooq

**Affiliations:** 1 School of Civil Engineering and Transportation, South China University of Technology, Guangzhou, 510640 China; 2 State Key Laboratory of Subtropical Building Science, South China University of Technology, Guangzhou, 510640 China; 3 China Coal Technology & Engineering Group, Nanjing Design & Research Institute Co., Ltd, Nanjing, 210031 China; 4 Center of Excellence in Transportation Engineering, Pak Austria Facshhoule Institute of Applied Sciences and Technology, Khanpur Road, Haripur, Pakistan

**Keywords:** Hydrology, Natural hazards

## Abstract

Combined with the spatial data processing capability of the Geographic Information Systems (GIS), the Pan Jiazheng method is extended from two-dimensional (2D) to three-dimensional (3D), and a 3D landslide surge height calculation method is proposed based on grid column units. First, the data related to the landslide are rasterized to form grid columns, and a force analysis model of 3D landslides is established. Combining the vertical strip method with Newton's laws of motion, dynamic equilibrium equations are established to solve the surge height. Moreover, a 3D landslide surge height calculation expansion module is developed in the GIS environment, and the results are compared with those of the 2D Pan Jiazheng method. Comparisons showed that the maximum surge height obtained by the proposed method is 24.6% larger than that based on the Pan Jiazheng method. Compared with the traditional 2D method, the 3D method proposed in this paper better represent the actual spatial state of the landslide and is more suitable for risk assessment.

## Introduction

A landslide disaster refers to a disaster caused by the overall downhill slide of rock mass or soil mass under the action of gravity and is one of the main geological disasters in the world. Quick sliding speeds and long sliding distances of medium and large landslides result in massive losses of life and property every year. Among these, the reservoir bank landslide produces a bigger surge during its sliding into the water, and this causes great harm to the passing ships and the surrounding buildings, receiving much concern around the globe. There are many surging events caused by landslides in the world, such as the 100 m high surges triggered by the landslide in the Vajont Reservoir, Italy, in 1963, killing at least 2,000 people^1^; the red rock surges in Wushan, Chongqing, China in 2015^2^; and the Dayantang surges in Hubei, China in 2014^3^; all of which brought about large casualties and economic losses. Therefore, it is of vital significance to evaluate the surge wave hazard caused by the reservoir bank landslide.

Calculating the surge height is one of the key indexes to evaluate the surge hazard. The methods of calculating the surge height can mainly be divided into theoretical analysis method^4–8^, numerical simulation method^10–14^, and physical modelling method^15–17^. Among them, the Pan Jiazheng method in the theoretical analysis method is widely used in engineering applications because of its simple modelling processes, which has few requirements for engineers and high precision.

The Pan Jiazheng method originated from Noda. Noda^4^ proposed an approximate method to find the amplitude of the largest surge in the nonlinear region by utilizing the solutions obtained from linear theory. Since then, many scholars have conducted more in-depth research. On this basis, Academician Pan Jiazheng^5^ divided the landslide body into many two-dimensional (2D) vertical strips and calculated the surge height by considering the horizontal and vertical movement of the landslide. This method is called the Pan Jiazheng method. The method has been applied and improved over the years. For example, Dai et al.^6^ used Pan Jiazheng method to calculate the sliding speed of Xiaduling landslide in the Three Gorges Reservoir area. Huang et al.^7^ improved the Pan Jiazheng method by considering the resistance of water and the change in the friction coefficient. Miao et al.^8^ proposed a sliding block model based on the 2D vertical strip method to predict the maximum surge height.

Although the Pan Jiazheng method has made some improvements, it is still in the 2D stage, and the calculation results will be different if the 2D section is selected. However, the actual state of the landslide is three-dimensional (3D), and the 2D analysis method cannot simulate the real landslide state in a reasonable way. Hu^18^ proposed that the value obtained by 2D state analysis is about 70% of the 3D state value. The Pan Jiazheng method based on 2D model has been unable to obtain high-precision result values, so the study of the Pan Jiazheng method based on 3D state analysis is of great significance to improve its calculation accuracy.

Geographic Information Systems (GIS) is widely used in geotechnical engineering^19–21^. GIS features strong spatial analysis capabilities. It can carry out unified management and storage of spatial data, with functions such as spatial positioning, spatial database management, digital elevation model establishment, etc. And it can provide users with multi-factor spatial analysis, prediction and forecasting, simulation optimization, and other analysis methods of spatial data. Its biggest feature is that it can transform vector data into raster dataset based on grid column unit model. Because of its good 3D spatial data processing capability, many scholars have added geotechnical professional models to their geographic information systems^22,23^. Xie's team^24–26^ built a 3D limit equilibrium method in the GIS, and developed the slope stability analysis module. This paper, based on the previous study, will establish a set of 3D landslide surge height calculation methods grounded in the GIS.

Coinciding with the spatial data processing capability of the GIS, this paper extends Pan Jiazheng's method from 2 to 3D and proposes a 3D landslide surge height calculation method based on the GIS. Firstly, considering the 3D spatial relation of grid column units, the calculating expression of the required parameters is given. Through the force analysis of the grid column unit, combined with Newton's law of motion and considering the action of water, the dynamic equation based on the grid column unit is established, and the sliding speed of the sliding body during the sliding process is solved, and then the surge height is calculated. At the same time, an expansion module for calculating surge height is developed based on Component Object Model (COM) technology in the ArcGIS environment, and the calculation is carried out through the case of the Kaiding landslide of Houziyan Hydropower Station, and compared with the results of 2D Pan Jiazheng method, which verified the applicability of the module.

After this introduction, the second section introduces the GIS-based 3D landslide surge height calculation method, gives the specific surge height calculation formula, and introduces the secondary development of the extension module. The third section verifies the correctness and applicability of the extension module through case calculation. Finally, the fourth section comes to discussion and summary.

## GIS-based method of calculating the surge height

### Grid column unit model based on GIS

**Figure 1:**
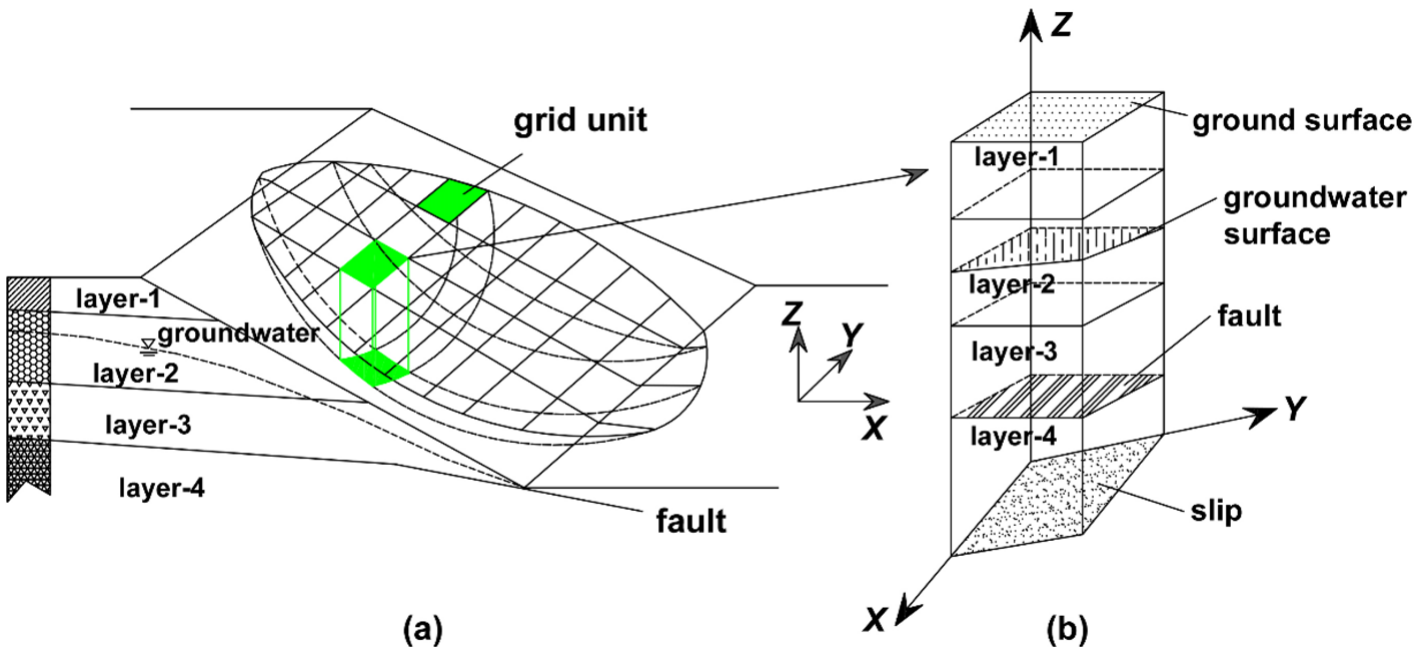
3D space model of landslide ((**a**) 3D schematic diagram of landslide, (**b**) 3D schematic diagram of one column).

For a slope, the representation of data is mainly in the form of vectors. These data include but are not limited to slip surface, strata, groundwater, fault, slip, and other types of data. These vector data layers can be converted to raster data layers using the spatial analysis capabilities of GIS to form a grid data set. The grid data structure consists of rectangular units. Each rectangular unit has a corresponding row and column number and is assigned an attribute value that represents the grid unit^27–31^. Therefore, the slope can be divided into square columns based on the grid units to form a grid column unit model, as shown in Fig. 1.

### The spatial relationships between parameters

**Figure 2:**
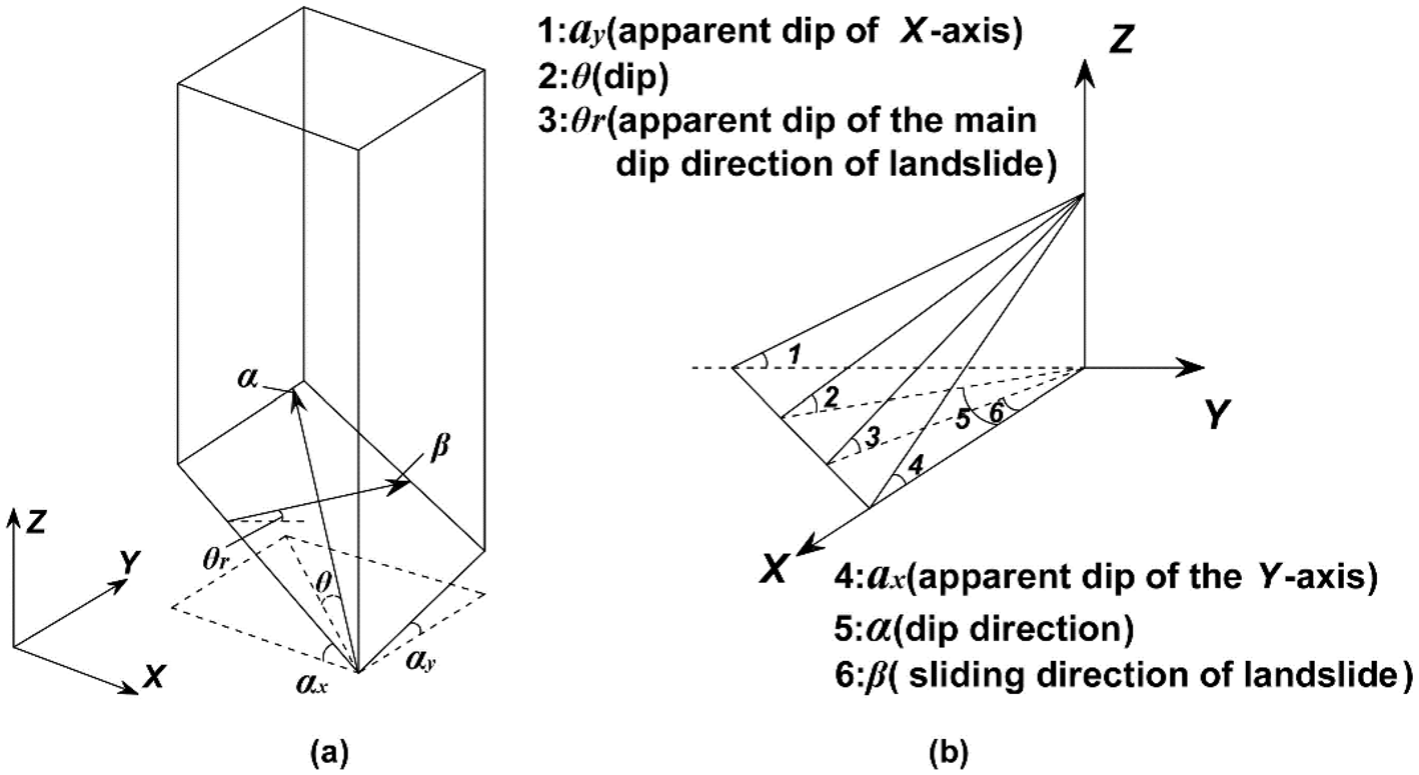
The spatial relationship between parameters. (**a**) Is the spatial relationship diagram of each parameter in the grid column; (**b**) is the calculation relationship diagram between parameters.

Figure 2 shows the spatial relationship between parameters. *θ* is the dip of the grid column at the slip surface; *α* is the dip direction of the grid column at the slip surface; *β* is the sliding direction of the landslide; *θ*_*r*_ is the apparent dip of the main inclination direction of the landslide; *α*_*x*_ is the apparent dip of the *X*-axis; and *α*_*y*_ is the apparent dip of the* Y*-axis. Of these six parameters, *α*, *θ*, and *β* are known and is calculated in a paper published by myself^25^. Other three parameters *α*_*x*_, *α*_*y*_ and *θ*_*r*_ can be calculated according to the spatial relationship in Fig. 2b, which are calculated as follows.

1 $$\alpha x = \arctan (\cos \alpha \tan \theta ),\alpha y = \arctan (\sin \alpha \tan \theta )$$

2 $$\theta_{r} = \arctan \left\{ {\tan \theta } \right.\left. {\left| {\cos \left( {\alpha - \beta } \right)} \right|} \right\}$$

The bottom area of one grid column is calculated by

3 $$A = cellsize^{2} \left[ {\frac{{\sqrt {\left( {1 - \sin^{2} \alpha x\sin^{2} \alpha y} \right)} }}{\cos \alpha x\cos \alpha y}} \right]$$

where *cellsize* represents the length of the side of each grid column.

The weight *W* of the grid column is expressed as

4 $$W = cellsize^{2} \sum\limits_{m = 1}^{n} {h_{m} r_{m} }$$

where *m* is the number of strata,* h*_*m*_ is the height of each stratum, and *r*_*m*_ is the unit weight of each stratum. For the grid column units above the water, *r*_*m*_ is calculated from the natural unit weight. For grid column units under water, *r*_*m*_ is calculated from the buoyant unit weight.

The pore water pressure is obtained as follows^32^.

5 $$u = R/{\text{cos}}\theta$$

where *R* is the distance from the centre bottom of the grid column to the water surface.

When the sliding body enters the water, the resistance of the water is calculated as follows^32^.

6 $$G{ = }\frac{1}{2}c_{w} \rho_{f} v^{2} S$$

where *G* is the resultant force of the resistance of the water to the sliding body (mN); *c*_*w*_ is the viscous resistance coefficient, which is 0.15 to 0.18; ^*ρ*^_*f*_ is the buoyant density (g/m^3^), taking the average of all stratum; *v* is the velocity of the landslide (m/s); and *S* is the surface area of the grid column in the water (m^2^).

### Dynamic equation based on grid column units

**Figure 3:**
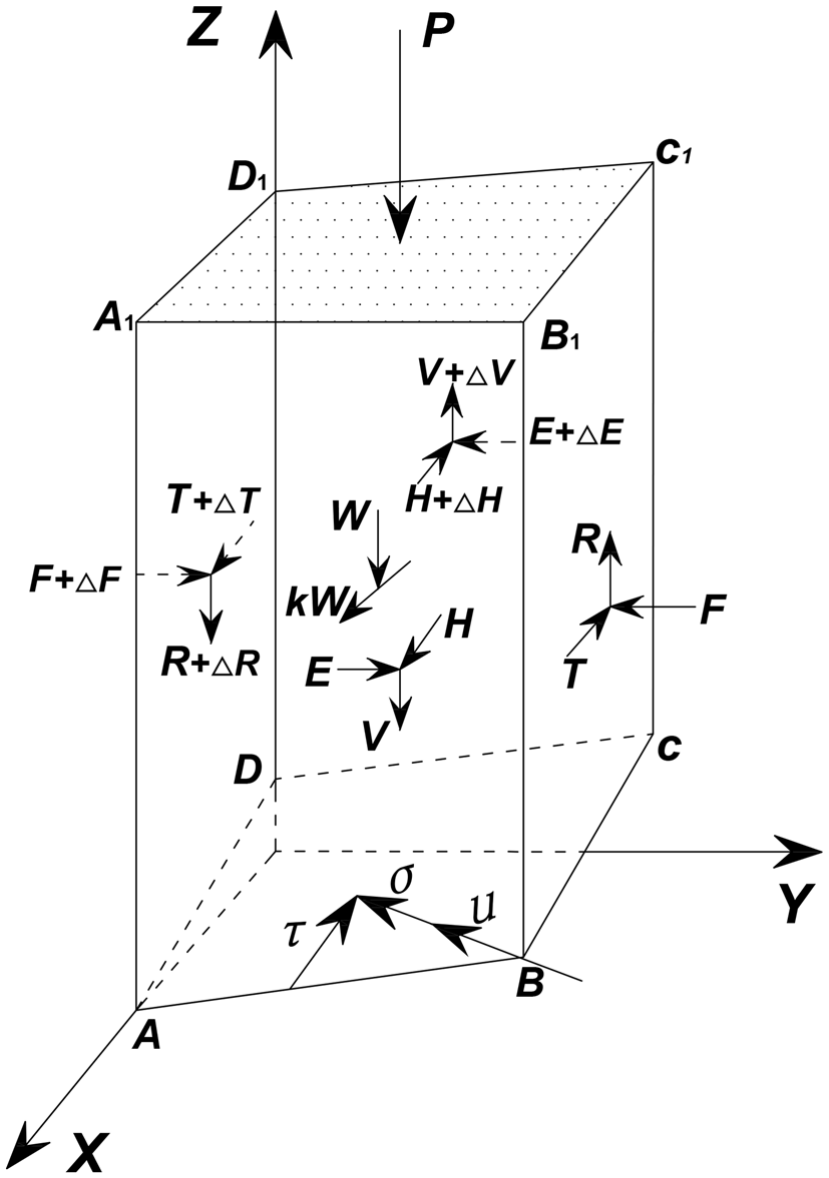
Force analysis of one grid column.

As shown in Fig. 3, it is the force analysis of one grid column. In the 3D sliding body, one random grid column *ABCDA*_1_*B*_1_*C*_1_*D*_1_ is selected, and the force analysis is explained as follows^26^.

1. The weight of one grid column is *W*; the direction is the *Z*-axis; and the weight acts at the centroid of the grid column.
2. The resultant horizontal seismic force is *kW*, where *k* is the “seismic coefficient”; the direction of *kW* is the sliding direction of the landslide; and the resultant horizontal force acts at the centroid of the grid column.
3. The external loads on the ground surface are represented by *P*; the direction of *P* is the *Z*-axis, and these external loads act at the centre of the top of the grid column. The external loads represent loads caused by objects on the surface of the landslide, such as buildings, trees, and so on.
4. The normal and shear stresses on the slip surface are represented by *σ* and *τ*, respectively. The normal stress is perpendicular to the slip surface, and the shear stress is in the sliding direction of the landslide. The normal and shear stresses act at the centre of the bottom of the grid column.
5. The pore water pressure on the slip surface is *u*. The direction of *u* is directed as *σ.*
6. The horizontal tangential forces on the vertical face at *y* = 0 and vertical face at *y* = *△y* (*△y* represents the size of the grid column along *Y*-axes) are *T* and *T* + *△T*, respectively; the vertical tangential forces on the vertical face at *y* = 0 and vertical face at *y* = *△y* are *R* and *R* + *△R*, respectively; the normal forces on the vertical face at *y* = 0 and vertical face at *y* = *△y* are *F* and *F* + *△F*, respectively; the horizontal tangential forces on the vertical face at *x* = 0 and vertical face at *x* = *△x* are *E* and *E* + *△E*, respectively; the vertical tangential forces on the vertical face at *x* = 0 and vertical face at *x* = *△x* are *V* and *V* + *△V*, respectively; and the normal forces on the vertical face at *x* = 0 and vertical face at *x* = *△x* are *H* and *H* + *△H*, respectively. For convenience, the resultant force between columns in the sliding direction of the landslide is defined as Δ*D*.

We assume that all the grid column units move continuously, do not separate in the macroscopic dimension and remain vertical after sliding, as also assumed by Pan Jiazheng^5^. The force analysis of one grid column and the spatial relationships between parameters at the slip surface are shown in Figs. 2 and 3, respectively.

**Figure 4:**
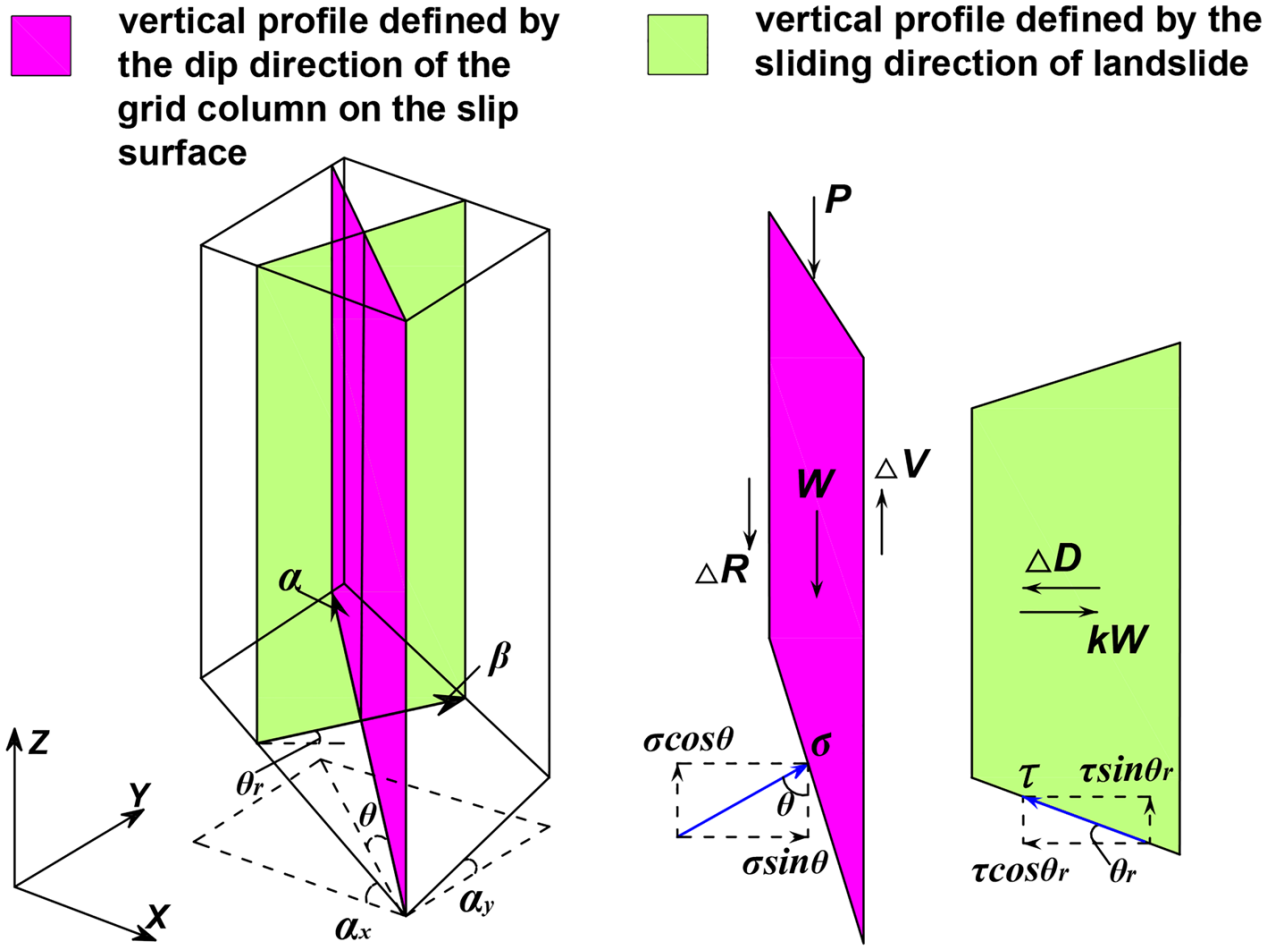
Force analysis in the vertical direction and sliding direction of the landslide.

We arbitrarily selected a grid column unit (the grid column unit in row *i* and column *j*). According to Newton’s laws of motion, dynamic equilibrium equations are established in the sliding direction of the landslide and the vertical direction. The force analyses in the sliding direction of the landslide and vertical direction are shown in Fig. 4.

7 $$A_{i,j} \tau_{i,j} \cos \theta r_{i,j} - A_{i,j} \sigma_{i,j} \sin \theta_{i,j} \cos \left( {\alpha_{i,j} - \beta } \right) - kW_{i,j} + \Delta D_{i,j} - G_{i,j} = \frac{{W_{i,j} }}{g}a_{x}$$

8 $$A_{i,j} \tau_{i,j} \sin \theta r_{i,j} + A_{i,j} \sigma_{i,j} \cos \theta_{i,j} - W_{i,j} - P_{i,j} + \Delta V_{i,j} - \Delta R_{i,j} = \frac{{W_{i,j} }}{g}ay_{i,j}$$

9 $$\tau_{i,j} = c_{i,j} + \left( {\sigma_{i,j} - u_{i,j} } \right)\tan \varphi_{i,j}$$

where *a*_*x*_ and *a* are the horizontal acceleration and vertical acceleration of the grid column, respectively; *φ* is the effective friction angle of the grid column at the slip surface; *g* is gravitational acceleration; *c* is the effective cohesion of the grid column at the slip surface.

According to this assumption, the horizontal acceleration *a*_*x*_ of each grid column unit is the same, and the vertical acceleration* a* of each grid column unit varies. Pan Jiazheng^5^ suggested that there is a certain proportional relationship between *a*_*x*_ and* a*, that is, *aa*_*x*_ = tan*δ*. *δ* is the horizontal inclination angle of the line connecting the centre bottom of the grid column to the centre bottom of the next grid column in the sliding direction of the landslide. The effect of vertical tangential forces is ignored, namely, Δ*V*-Δ*R* = 0; therefore, Eq. (8) can be transformed as follows.

10 $$A_{i,j} \tau_{i,j} \sin \theta r_{i,j} + A_{i,j} \sigma_{i,j} \cos \theta_{i,j} - W_{i,j} - P_{i,j} = \frac{{W_{i,j} }}{g}ax\tan \delta_{i,j}$$

The simultaneous Eqs. (9) and (10) can be obtained as follows.

11 $$\sigma_{i,j} = \frac{{A_{i,j} \sin \theta r_{i,j} \left( {u_{i,j} \tan \varphi_{i,j} - c_{i,j} } \right) + W_{i,j} + P_{i,j} + \frac{{W_{i,j} }}{g}ax\tan \delta_{i,j} }}{{A_{i,j} \left( {\sin \theta r_{i,j} \tan \varphi_{i,j} + \cos \theta_{i,j} } \right)}}$$

For the entire sliding body, the forces between the grid columns are internal forces, that is, the resultant force is 0, yielding Eq. (12).

12 $$\sum\limits_{I} {\sum\limits_{J} {\Delta D_{i,j} = 0} }$$

By summing all the grid column units, the horizontal acceleration *a*_*x*_ can be determined by Eq. (7).

13 $$a_{x} = \left[ {\sum\limits_{I} {\sum\limits_{J} {\frac{{A_{i,j} \tau_{i,j} \cos \theta r_{i,j} - A_{i,j} \sigma_{i,j} \sin \theta_{i,j} \cos \left( {\alpha_{i,j} - \beta } \right) - kW_{i,j} { - }G_{i,j} }}{{W_{i,j} }}} } } \right]g$$

Substituting Eqs. (9) and (11) into Eq. (13) yields the following equation.

14 $$a_{x} = \left[ {\sum\limits_{I} {\sum\limits_{J} {\frac{{B_{i,j} + E_{i,j} + F_{i,j} \left( {W_{i,j} + P_{i,j} } \right) - (kW_{i,j} + G_{i,j} )L_{i,j} }}{{W_{i,j} (L_{i,j} - \tan \delta_{i,j} F_{i,j} )}}} } } \right]g$$

where

15 $$B_{i,j} = A_{i,j} \cos \theta r_{i,j} \cos \theta_{i,j} \left( {c_{i,j} - u_{i,j} \tan \varphi_{i,j} } \right)$$

16 $$E_{i,j} = A_{i,j} \cos \left( {\alpha_{i,j} - \beta } \right)\sin \theta r_{i,j} \sin \theta_{i,j} \left( {c_{i,j} - u_{i,j} \tan \varphi_{i,j} } \right)$$

17 $$F_{i,j} = \left[ {\cos \theta r_{i,j} \tan \varphi_{i,j} - \sin \theta_{i,j} \cos \left( {\alpha_{i,j} - \beta } \right)} \right]$$

18 $$L_{i,j} = \sin \theta r_{i,j} \tan \varphi_{i,j} { + }\cos \theta_{i,j}$$

### Calculation of the sliding velocity

The steps in calculating the landslide sliding velocity are as follows.

**Figure 5:**
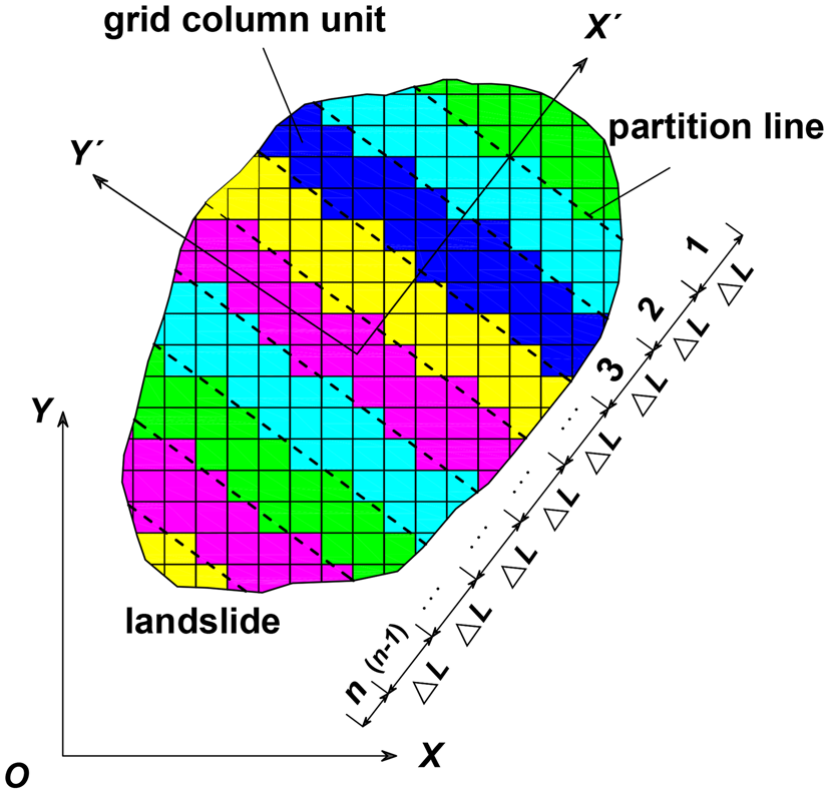
Rasterization and partitioning of landslides.

1. Using the spatial analysis capability of GIS, the landslide body is rasterized, and the size of the grid column unit (*i*, *j*) can be set to an arbitrary square. A partitioning line is drawn from the bottom to the top of the landslide every Δ*L* in the sliding direction of the landslide, and the resulting regions are numbered zone 1, zone 2, zone 3, …, zone (n-1), zone n. Each partition includes numbers of grid column units, and the length of zone n is less than or equal to Δ*L*, as shown in Fig. 5. For a grid column unit that is not completely contained within a partition, if the area within the partition is greater than half of the total area, the unit is divided into that partition; otherwise, the unit is divided into the next partition.
2. For each grid column unit, the parameters required in Eq. (14) are calculated.
3. *t*_0_ is the starting point of when the landslide body begins to slide, and *t*_0_ = 0. When the landslide body moves distance Δ*L* sequentially in the sliding direction of the landslide, the corresponding time is recorded as *t*_1_, *t*_2_, *t*_3_…*t*_n_, and the corresponding velocity is expressed as *v*_x1_, *v*_x2_, *v*_x3_…*v*_xn_.
4. The horizontal acceleration at* t*_0_ can be calculated by Eq. (14) and is denoted as* a*_*x*0_, and the velocity at time *t*_0_ is zero. After sliding distance Δ*L* is reached, the following equations can be obtained.
  19 $$v_{x1} = \sqrt {2a_{x0} \Delta L}$$
  20 $$t_{1} = t_{0} + \sqrt {\frac{2\Delta L}{{a_{x0} }}}$$
5. At *t* = *t*_1_, the landslide body has horizontally moved by a distance Δ*L* in the sliding direction of the landslide, zone 1 has slipped from the sliding surface. The horizontal acceleration *a*_*x*1_ at *t*_1_ is still calculated by Eq. (14). Unlike* t*_0_, the weight for zone (n-1) changes to the weight for zone n, and the weight for zone (n-2) becomes the weight for zone (n-1), and so on (At this time, there is no grid column for zone n). After* a*_*x*1_ is calculated, the following can be established.
  21 $$v_{x2} = \sqrt {2a_{x1} \Delta L + v_{x1}^{2} }$$
  22 $$t_{2} = t_{1} + \frac{{v_{x2} - v_{x1} }}{{a_{x1} }}$$
6. The calculation is continued in turn. When the obtained horizontal acceleration is negative, the maximum velocity can be obtained. Finally, *a*_*x*_ and* v*_*x*_ in the calculation process can be plotted as respective curves versus the sliding time.

### Surge height calculation

The China Institute of Water Resources and Hydropower Research proposed an empirical formula for surge height calculation^33^. In the formula, the main factors that affect the surge height are the sliding velocity and volume of the landslide. The formula for calculating the maximum surge height is as follows.

23 $$\xi_{\max } = d\frac{{v_{m}^{1.85} }}{2g}V^{0.5}$$

where *ξ*_max_ is the maximum surge height (m); *d* is the comprehensive influence coefficient, with an average value of 0.12; *v*_*m*_ is the maximum sliding velocity (m/s); *V* is the volume of the landslide body in the water (m^3^); and *g* is gravitational acceleration, which equals 9.8 m/s^2^.

The formula for calculating the surge height at different distances from the landslide body is as follows.

24 $$\xi = d_{1} \frac{{v_{m}^{n} }}{2g}V^{0.5}$$

where *ξ* is the surge height at a point from the landslide body *L* metres (m); *n* is the calculation coefficient, which is 1.4; and *d*_1_ is the influence coefficient related to distance *L*, which is determined by the following formula.

25 $$d_{1} = \left\{ \begin{gathered} 0.5{ , }\,\left( {L \le 35} \right) \, \hfill \\ 6.1274L^{ - 0.5945} {, }\,\left( {L > 35} \right) \hfill \\ \end{gathered} \right.$$

### Program implementation

Combined with the surge height calculation method, an expansion module was developed based on component object model (COM) technology in the ArcGIS environment^25,26^.

**Figure 6:**
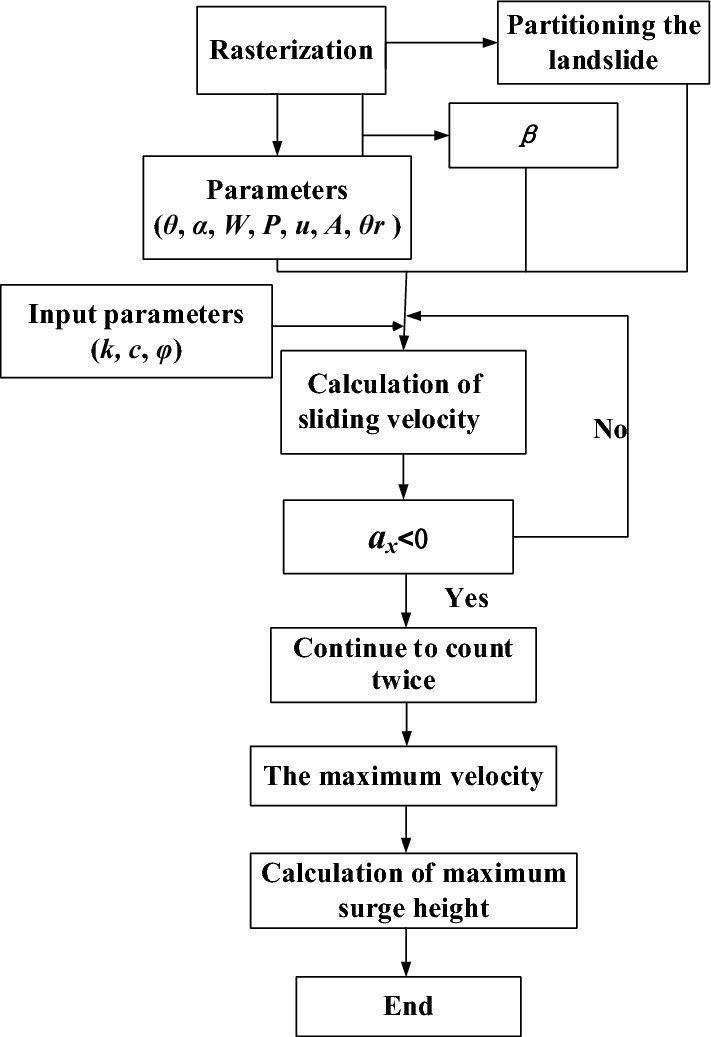
The algorithmic steps.

First, rasterize the data related to slope in ArcGIS software to form a raster data set (including the information of elevation, strata, groundwater, fault, slip surface, etc.), and calculate the required parameters by using the extension module. Then, divide the sliding body into units, and calculate the sliding speed at each time point step by step with the extension module. And the calculation stops when the acceleration is less than 0. Finally, obtain the maximum sliding speed and calculate the maximum surge height. Fig. 6 illustrates the algorithmic steps.

## Case study and discussion

### Overview of the project

**Figure 7:**
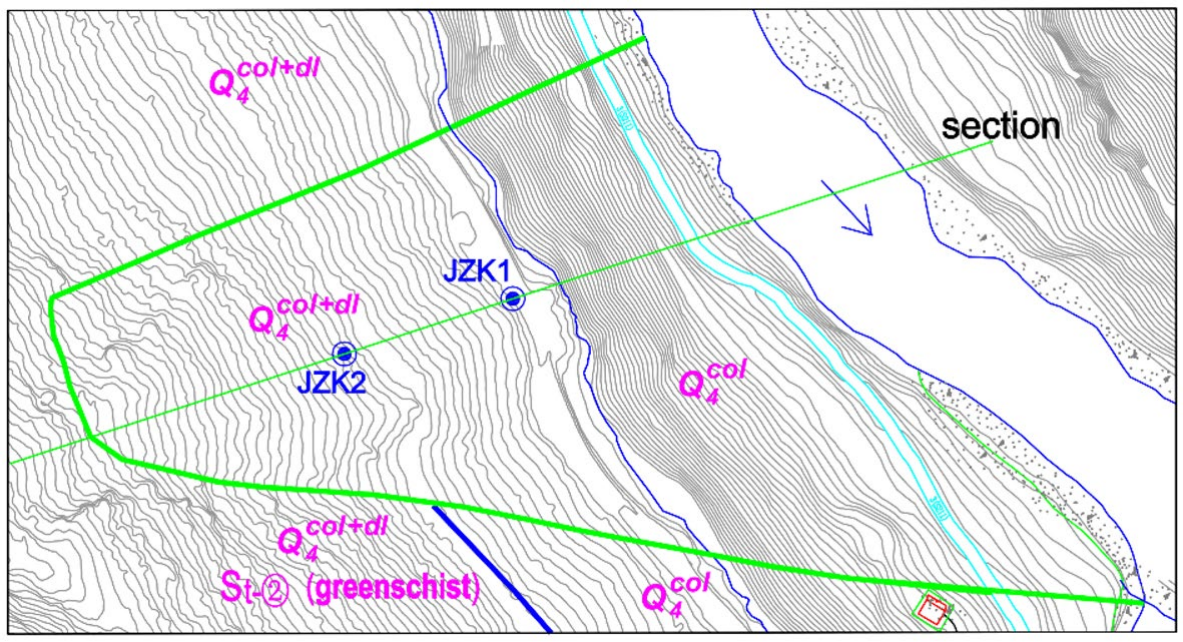
The plan view of the Kaiding landslide.

**Figure 8:**
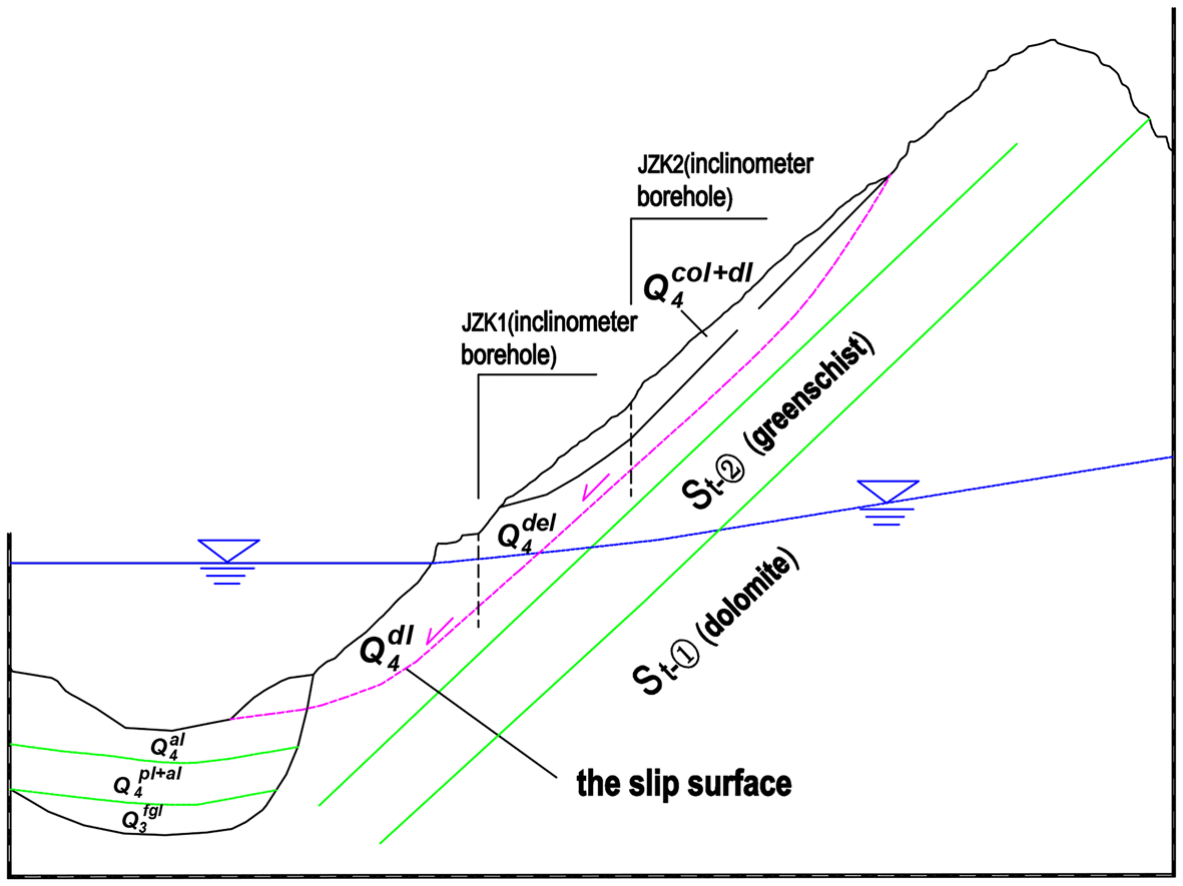
The section view of the Kaiding landslide.

The Kaiding landslide is approximately 14.5 km away from the dam of the Houziyan hydropower station in Sichuan, China. The length of the landslide along the river is approximately 490 m, the top elevation is 2080 m, the bottom elevation is 1754 m, and the volume is approximately 4.5 million m^3^. Plan and section views are shown in Figs. 7 and 8, respectively. The scale in Fig. 7 is 1:50,000, and the river direction is north to south.

### Calculation of the sliding velocity

**Figure 9:**
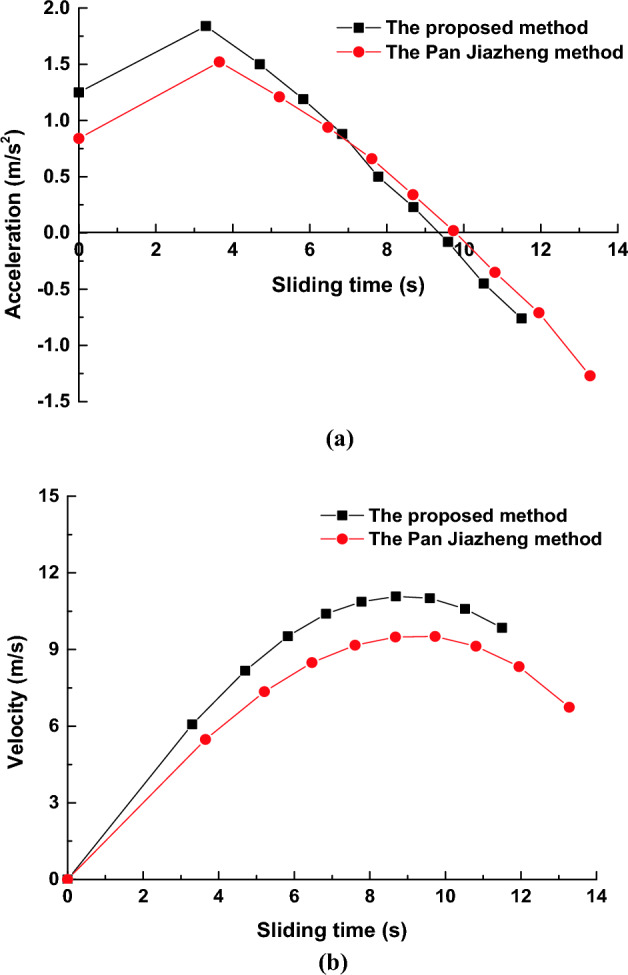
(**a**) Horizontal acceleration curve with the sliding time. (**b**) Sliding velocity curve with the sliding time.

The unit size of a grid column is 5 m × 5 m, and Δ*L* = 10 m. The internal friction angle *φ* at the slip surface is 22.8°, the natural unit weight is 18.84 kN/m^3^, the buoyant unit weight is 19.43 kN/m^3^, the buoyant density is 2.11 × 10^6^ g/m^3^, the viscous resistance coefficient is 0.18, and the elevation of the reservoir water level is 1810.3 m. When the landslide body slides, the effective cohesion *c* at the slip surface will decrease to 0, that is, *c* = 0^5^. Using this method and Pan Jiazheng's 2D method, the acceleration and velocity curves with the sliding time can be obtained, as shown in Fig. 9a,b, respectively. The calculation results are shown in Table 1.

**Table 1:** Calculation results.

The Pan Jiazheng method			The proposed method		
*t*(s)	*a*_*x*_(m/s^2^)	*v*_*x*_ (m/s)	*t*(s)	*a*_*x*_(m/s^2^)	*v*_*x*_ (m/s)
0	0.84	0	0	1.25	0
3.65	1.52	5.48	3.30	1.84	6.07
5.21	1.21	7.35	4.70	1.50	8.17
6.47	0.94	8.49	5.83	1.19	9.52
7.61	0.66	9.17	6.84	0.88	10.40
8.68	0.34	9.49	7.78	0.60	10.96
9.73	0.02	9.51	8.67	0.28	11.21
10.81	− 0.35	9.13	9.57	− 0.08	11.14
11.95	− 0.71	8.33	10.48	− 0.45	10.73
13.28	− 1.27	6.74	11.45	− 0.90	9.86

The calculation results indicate that the maximum velocity obtained by the proposed method is 11.21 m/s, the starting acceleration is 1.25 m/s^2^, and the sliding time required to reach the maximum velocity is 8.67 s. In comparison, the maximum velocity obtained by the Pan Jiazheng method is 9.51 m/s, the starting acceleration is 0.84 m/s^2^, and the sliding time required to reach the maximum velocity is 9.73 s.

Comparing the results of the proposed method with those of the Pan Jiazheng method, the maximum velocity of the proposed method is 15.2% higher than that calculated by the Pan Jiazheng method, the starting acceleration is 32.8% higher, and the sliding time required to reach the maximum velocity is 1.06 s short.

### Surge height calculation

According to the most dangerous working conditions, it is assumed that the landslide body all slips into the water. The volume *V* of the landslide body under water is 340 × 10^4^ m^3^. According to Eqs. (25) and (26), the maximum surge height obtained by the proposed method is 9.66 m, and the surge height at the dam site is 0.56 m. The maximum surge height obtained by the Pan Jiazheng method is 7.28 m and the surge height at the dam site is 0.44 m. The calculation results are shown in Table 2.

**Table 2:** Calculation results.

	The Pan Jiazheng method	The proposed method
The surge height at the dam site (m)	0.44	0.56
The maximum surge height (m)	7.28	9.66

The landslide is approximately 14.5 km from the dam, the crest elevation is 1847.02 m, and the elevation of the reservoir water level is maintained at 1810.3 m. When the surge height at the dam site is 0.56 m, water will not flow over the dam crest and the safe operation of the dam will not be affected.

The maximum surge height obtained by the proposed method is 24.6% larger than that based on the Pan Jiazheng method, and the surge height at the dam site obtained by the proposed method is 21.4% larger than that based on the Pan Jiazheng method.

## Discussion and conclusions

### Discussion

The calculation results show that the difference between the results of the 2D method and the 3D method is 24.6%. Hu^13^ proposed that the value obtained by 2D state analysis is about 70% of the 3D state value. The result is consistent with Hu's prediction. Compared that of the 2D method, the computational model of the 3D method better represents the actual spatial state of the landslide. Therefore, the method in this paper is more applicable than Pan Jiazheng method in actual risk evaluation.

This paper proposes a 3D landslide surge height calculation method, and it divides the landslide into several grid column units. The method assumes that all the grid column units move continuously and do not separate in the macroscopic dimension, remaining vertical after sliding. That is, assume that the column units are rigid materials. But in the actual sliding process, the column units are unable to keep vertical regularly, especially in the soil landslide, while for the rock landslide, its column units can keep better integrity during the sliding process, so the method in this paper will have relatively good applicability for the rock landslide.

### Conclusions

Combined with the spatial data processing capability of the GIS, the Pan Jiazheng method is extended from 2 to 3D, and a 3D landslide surge height calculation method is proposed for the first time. Combined with Newton's law of motion, the dynamic balance equation for calculating the sliding speed of a 3D sliding body is derived, and then the surge height is calculated.

This is the first time to combine the surge height calculation model with GIS. At the same time, an extension module is developed based on ArcGIS software, and the feasibility of the module is verified by a case study. The module has the advantages of unified data format and simple preparation process.

Because the Pan Jiazheng method is a calculation method focused on 2D sections, the calculation results will be different if different sections are selected. After the 3D landslide is carried out in rasterization, the 3D calculation model based on the grid column unit is established in this paper, which overcomes the above defects and makes the calculation model close to the actual situation, therefore the method in this paper is more applicable than Pan Jiazheng method in actual risk evaluation.

As the application of GIS in geotechnical engineering becomes increasingly extensive, the calculation method of surge height established in GIS in this paper will provide a theoretical basis for scholars to add surge height calculation modules in their respective geographic information Systems.

## Supplementary Information

Supplementary Information.

## Data Availability

The datasets used and/or analysed during the current study available from the corresponding author on reasonable request.
