# Supplementary material for: GIS-based calculation method of surge height generated by three-dimensional landslide
Source: Sci Rep. 2023 May 11;13:7684. doi: 10.1038/s41598-023-34798-1 (PMC10175286; doi:10.1038/s41598-023-34798-1)
Supplement: Supplementary file 1 — Supplementary Information. [file 41598_2023_34798_MOESM1_ESM.pdf]

GIS-based calculation method of surge height generated by three-dimensional landslide

Guo Yu[1], Xiaowen Zhou [2,*], Lei Bu [3], Chengfeng Wang[1], and Asim Farooq [4]

[1]School of Civil Engineering and Transportation, South China University of Technology., Guangzhou 510640, China.

[2]State Key Laboratory of Subtropical Building Science, South China University of Technology, Guangzhou 510640, China.

[3]China Coal Technology & Engineering Group Nanjing Design & Research Institute Co., Ltd, Nanjing 210031, China.

[4] Center of excellence in transportation engineering, Pak Austria Facshhoule institute of applied sciences and technology, Khanpur Road, haripur, Pakistan.

[*] Corresponding author: Xiaowen Zhou (xwzhou@scut.edu.cn)

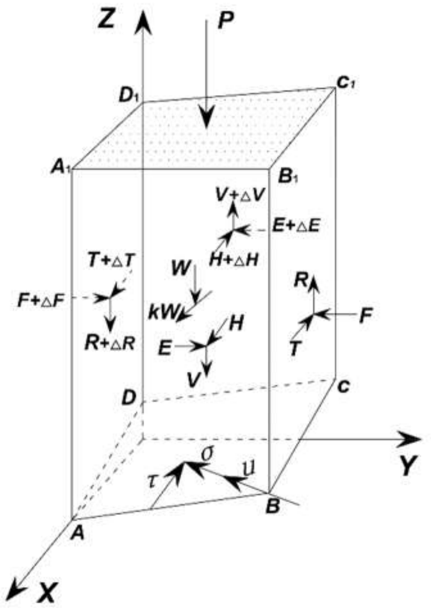

Fig. 3. Force analysis of one grid column.

(1) The weight of one grid column is *W*; the direction is the *Z*-axis; and the weight acts at the centroid of the grid column.

(2) The resultant horizontal seismic force is *kW*, where *k* is the "seismic coefficient"; the direction of *kW* is the sliding direction of the landslide; and the resultant horizontal force acts at the centroid of the grid column.

(3) The external loads on the ground surface are represented by *P*; the direction of *P* is the *Z*-axis, and these external loads act at the centre of the top of the grid column.

The external loads represent loads caused by objects on the surface of the landslide, such as buildings, trees, and so on.

(4) The normal and shear stresses on the slip surface are represented by σ and τ, respectively. The normal stress is perpendicular to the slip surface, and the shear stress is in the sliding direction of the landslide. The normal and shear stresses act at the centre of the bottom of the grid column.

(5) The pore water pressure on the slip surface is u. The direction of u is directed as σ.

(6) The horizontal tangential forces on the vertical face at y=0 and vertical face at y=dy (dy represents the size of the grid column along Y-axes) are T and T+dT, respectively; the vertical tangential forces on the vertical face at y=0 and vertical face at y=dy are R and R+dR, respectively; the normal forces on the vertical face at y=0 and vertical face at y=dy are F and F+ dF, respectively; the horizontal tangential forces on the vertical face at x=0 and vertical face at x=dx are E and E+ dE, respectively; the vertical tangential forces on the vertical face at x=0 and vertical face at x=dx are V and V+ dV, respectively; and the normal forces on the vertical face at x=0 and vertical face at x=dx are H and H+ dH, respectively. For convenience, the resultant force between columns in the sliding direction of the landslide is defined as ΔD.

References

Yu G, Xie M, Liang J, et al. A GIS-based 3D slope stability analysis method based on the assumed normal stress on the slip surface[J]. Scientific Reports, 2020, 10(1): 291-298.
